# 

**Published:** 1893-03-18

**Authors:** 


					18,1893. THE HOSPITAL,
Ww, CONTENTS.
Wlj^f j
3 *5? Pension Fund Doing"? ?
387
?round%lae,?ension Fund Doing"? ?
fcedir*! Hospitals . . - .
Medical ?r? ?osPifals
? WiTiTJ?1?tory fror
from the Earliest Times.
By E. T.
^ y lrom Ea
*easW? *T0N? M.b. Oxon.
rliest .
XLYII.
-Paraceleus -
peuSio??G,T0N' M-A > M
?leti2n^for Ola Age.
B. O:
II.-
:on. XLVII.?Paracelsus
Essential Points
S90
Dieti^s 10r Ol
Com^Pisease.
Age. II.? Essential ]
By Mr?. Ken est H&kt.
'oints
XVI.-
-Diabetes
[eon.)
391
Co&teiyi>T se" ^RN
^o^?.raty Theory and ]
est Hart. XVI.?Diabetes (eon.)
Research
892
4?J
^ Qln.fi ???
A Piasrue of Specialises"?How to Catch Rhen-
Bacteria
uy ui opociuiiai-H ?nuw lu v/al
393
??tes a^orR Bact?"ia
Scra-n^ ?ew& ?
394
scrar>B r5 eWB ?
?he Bn^i?^G1?anil38's
402
Bft.i iyleanings
I'he at,?^^orld of Medi
medicine and Science
The Stnrt? *orld of Medicine and Science
otudent of Medicine.
The Hospital Clinic?
Londos Hospital.-
-The Treatment of Ohorea
395
The Hospital foe Sick Children, Great Ormond Street.?
Treatment of Inr.nsausception
S95
The Bat at Eye Infirmary.-
-The Treatment of Headaches
Bristol Children's Hospital.-
-The Treatment of Acate Rhea-
matism -tnd Its Oompl cationa
S97
New Drugs, Appliances, and Things Medical.?
Ohristia
The Institutional workshop?
Hospital Administration.-
-Outpost Hospitals in Large Towns
399
.PRACTICAL JJKPAETMENTS.-
-Hospital Dresser s Table -
Hospitals op the world.-
-The Progress of English Nursing ?
401
EXTRA SUPPLEMENT.?THE NUESING MIRROR.
Ifew Jtujs.'1'iiA s u jf r Jj .u m
Nursing" World.?Princess Victoria of
Thfi p 1S\"H?lB(eiu?1The Nnrse Dolls?A Modest Request?
Mit.,^8lIreeH pp tal?The L f of the Ward?Olapham
at T . y Hospital?From Wash-tub to Ward?Liberality
Enla^Eoa8ter?D fficnlt Nursing?Sick and Hungry?
Patriand Improvement? Banished Bsds ? St.
? Bosr,if iS.. J?rap~^llort Items ?Tho Disabled Nurse?" The
Endowed Bed
of ConsuniPt^on- VI.?The Temperature
clxxxi
clxxxiii
Minor Appointments-Derby County Asylum . - clxxxiii
Tlie Boyal National Pension Pom for ffun-es ? - clxxxiii
London Hospital Training1 School for Purses- - o!xxxv
Night Nursing?Everybody's Opinion.?Portable Tank clxxxvi
Notes and Queries clxxxvi
Our Examination Questions-Hints for Nurses ? clxxxvii
For Beading to the Sick.?Quickening?Appointment clxxxvii
The Muses' Lookiug Glass ? ? - - - -clxxxviii
Death in our Banks -Wants and Workers - ? clxxxviii
The Book World for Women and Nurses ? ? - clxxxix

				

